# Effects of Addition of Linseed and Marine Algae to the Diet on Adipose Tissue Development, Fatty Acid Profile, Lipogenic Gene Expression, and Meat Quality in Lambs

**DOI:** 10.1371/journal.pone.0156765

**Published:** 2016-06-02

**Authors:** Olaia Urrutia, José Antonio Mendizabal, Kizkitza Insausti, Beatriz Soret, Antonio Purroy, Ana Arana

**Affiliations:** Escuela Superior de Ingenieros Agrónomos, Departamento de Producción Agraria, Universidad Pública de Navarra, Pamplona, Spain; Wageningen UR Livestock Research, NETHERLANDS

## Abstract

This study examined the effect of linseed and algae on growth and carcass parameters, adipocyte cellularity, fatty acid profile and meat quality and gene expression in subcutaneous and intramuscular adipose tissues (AT) in lambs. After weaning, 33 lambs were fed three diets up to 26.7 ± 0.3 kg: Control diet (barley and soybean); L diet (barley, soybean and 10% linseed) and L-A diet (barley, soybean, 5% linseed and 3.89% algae). Lambs fed L-A diet showed lower average daily gain and greater slaughter age compared to Control and L (*P <* 0.001). Carcass traits were not affected by L and L-A diets, but a trend towards greater adipocyte diameter was observed in L and L-A in the subcutaneous AT (*P =* 0.057). Adding either linseed or linseed and algae increased α-linolenic acid and eicosapentaenoic acid contents in both AT (*P <* 0.001); however, docosahexaenoic acid was increased by L-A (*P <* 0.001). The *n*-6/*n*-3 ratio decreased in L and L-A (*P <* 0.001). Algae had adverse effects on meat quality, with greater lipid oxidation and reduced ratings for odor and flavor. The expression of lipogenic genes was downregulated in the subcutaneous AT (*P <* 0.05): a*cetyl-CoA carboxylase 1* (*ACACA*) in L and L-A and *lipoprotein lipase* (*LPL*) and *stearoyl-CoA desaturase* (*SCD*) in L-A. *Fatty acid desaturase 1* (*FADS1*), *fatty acid desaturase 2* (*FADS2*) and *fatty acid elongase 5 (ELOVL5*) were unaffected. In the subcutaneous AT, supplementing either L or L-A increased *peroxisome proliferator-activated receptor gamma* (*PPARG)* and *CAAT-enhancer binding protein alpha (CEBPA)* (*P <* 0.05), although it had no effect on *sterol regulatory element-binding factor 1* (*SREBF1*). In the intramuscular AT, expression of *ACACA*, *SCD*, *FADS1* and *FADS2* decreased in L and L-A (*P <* 0.001) and *LPL* in L (*P <* 0.01), but *PPARG*, *CEBPA* and *SREBF1* were unaffected.

## Introduction

Adipose tissue (AT) amount and fatty acid (FA) composition play major roles in meat quality in terms of sensory properties and health considerations [1]. Adipose tissue growth involves adipocyte hypertrophy and hyperplasia and the intensity of each process is age-dependent and depot-specific [2]. Adipogenesis is regulated by a complex network of transcription factors being *peroxisome proliferator-activated receptor gamma* (*PPARG)* and *CAAT-enhancer binding protein alpha* (*CEBPA)* the central regulators of this process, which is regulated by PUFA [3]. Long-chain *n*-3 PUFA (LCPUFA), particularly docosahexaenoic acid (DHA, C22:6 *n*-3) and eicosapentaenoic acid (EPA, C20:5 *n*-3), and the C18:2 *c-*9, *t-*11 isomer of CLA are known to be beneficial for human health [4]. Thus, vegetable oils or seeds containing α-linolenic acid (ALA, C18:3 *n*-3) have been studied to enrich meat with *n*-3 PUFA and enhance the endogenous synthesis of *n*-3 LCPUFA. Linseed addition has been found to be effective in increasing the ALA content of meat, however, there was no or was minor, effect on EPA, DHA and CLA [5,6] probably due to dietary PUFA inhibiting the expression of lipogenic genes and proteins involved in their synthesis [7,8]. Then, supplementary sources of EPA and DHA, such as marine algae, are being examined. Moreover, addition of PUFA to animal diets may affect AT growth and have adverse effects on meat quality due to its greater potential for oxidation and generation of off-flavors [9]. Following the hypothesis that addition of PUFA to the diet in lamb feeds could alter FA composition, AT development and lipogenic gene expression, this work aimed to study the effect of linseed or the effect of partial substitution of linseed with marine algae on AT development, FA profile, adipogenic and lipogenic genes expression in subcutaneous (SC) and intramuscular (IM) AT and meat quality in lambs.

## Material and Methods

### Ethics statement

Animal care, handling and experimental procedures were in compliance with relevant international guidelines (European Union procedures on animal experimentation—Directive 2010/63/EU) that regulate the protection of animals used for scientific purposes [10]. These define that in the case of experiments carried out under standard production conditions, no approval from an ethics committee is required. The slaughtering was performed at the commercial abattoir “La Protectora S.A.” in Pamplona (Spain, 42°49’13.07”N, 1° 40’ 51.65” W) following the European Union regulations (Council Regulation, EC, No 1099/2009) [11] that regulate the protection of animals at the time of killing. Therefore, animals included in this experiment were subjected to the same welfare conditions as production animals in farms and abattoir, and all efforts were made to minimize suffering.

### Animals, diets and tissue sampling

Thirty-three unrelated male Navarra breed lambs (11 lambs per group) were weaned at 16.3 ± 0.3 kg of body weight and 55.1 ± 1.5 d of age and allocated randomly to one of the three treatment groups: control group (C), concentrate composed of barley and soybean; linseed group (L), barley and soybean with 10% linseed (DM basis) supplied by Valomega 160 (Pinallet S.A., Cardona, Spain) consisting of 70% Tradi-LIN extruded linseed and 30% wheat bran; linseed and algae group (L-A), barley and soybean with 5% linseed (DM basis) and 3.89% marine microalgae (DM basis; Market DHA Gold, *Schizochytrium* spp.; Market Biosciences Corp., Columbia, MD, USA). Both linseed and marine algae in LA diet supplied the same amount of crude fat. The three diets were isoproteic and isoenergetic (Table 1).

**Table 1:** Ingredients, chemical composition and fatty acid composition of experimental diets.

Item^2^	Treatment^1^		
	C	L	L-A
Ingredient, % DM			
Barley	74.03	65.17	66.19
Soybean meal	22.37	16.94	19.20
Extruded linseed (Valomega 160)^3^	-	14.29	7.15
Marine microalgae (DHA-Gold)^4^	-	-	3.89
CaCO_3_	1.76	1.76	1.76
NaHCO_3_	1.00	1.00	1.00
NaCl	0.50	0.50	0.50
Mineral vitamin supplement^5^	0.30	0.30	0.30
Flavorings	0.04	0.04	0.04
Chemical composition, % DM			
CP	16.87	16.63	16.54
Crude fat	2.93	6.10	6.68
Crude fiber	4.35	4.63	4.30
Ash	5.23	5.54	5.77
Metabolizable energy, Mcal/kg	2.70	2.75	2.80
Fatty acid composition, g/100 g of total FAME			
C16:0	24.0	13.2	19.9
C18:0	3.29	3.20	2.60
C20:0	0.11	0.13	0.15
C18:1 *c-*9	12.1	15.8	11.1
C18:2 *c-*9, *t-*11 (CLA)	0.24	0.25	0.08
C18:2 *n*-6 (LA)	48.5	30.5	28.3
C18:3 *n*-6	0.05	0.17	0.16
C18:3 *n*-3 (ALA)	4.43	33.5	22.6
C20:5 *n*-3 (EPA)	0.08	0.04	0.34
C22:6 *n*-3 (DHA)	0.01	0.01	6.61

^1^Treatments: C = control; L = 10% linseed; L-A = 5% linseed with 3.89% marine algae.

^2^CP = crude protein; FAME = fatty acid methyl esters; LA = linoleic acid; ALA = α-linolenic acid; EPA = eicosapentaenoic acid; DHA = docosahexaenoic acid.

^3^Valomega 160 (Pinallet S.A., Cardona, Spain). Product consisted of 70% Tradi-LIN extruded linseed and 30% wheat bran.

^4^DHA-Gold (Market Biosciences Corp., Columbia, MD, USA). Algal meal high in DHA derived from *Schizochytrium* spp.

^5^Composition, content per kilogram: vitamin A (2,000,000 IU), vitamin D3 (550,000 IU), vitamin E (2000 mg), vitamin B2 (125 mg), vitamin B1 (125 mg), Mg (12,500 mg), Mn (6125 mg), Zn (9900 mg), I (100 mg), Fe (3300 mg), Cu (1100 mg), Co (150 mg), Se (25 mg), anti-rust (62.5 mg).

The lambs were reared in adjacent pens under the same environmental conditions and were given *ad libitum* access to concentrate feed throughout the experimental period. Lambs were weighed twice weekly in the morning before feeding and were slaughtered at common targeted body weight of 26.7 ± 0.3 kg, which is the commercial slaughter weight (24–28 kg LW) for this type of lambs. Animals were transported to the abattoir “La Protectora S.A.” (42°49’13.07”N, 1° 40’ 51.65” W) and immediately after slaughter, carcasses were weighed (hot carcass weight). Samples (5 g) of SC AT and IM AT of *Longissimuss dorsi* for RNA analysis were taken at the 10^th^ rib of the right carcass side with a scalpel blade, collected in hermetically sealed bags, snap frozen in liquid nitrogen and stored at -80°C. Samples of SC and IM AT (1 g) for adipocyte size determination were taken at the 10^th^ rib of the right carcass side and placed in glass vials containing 10 mL of Tyrode´s solution (0.15 *M* NaCl; 6 m*M* KCl; 2 m*M* CaCl_2_; 6 m*M* glucose; 2 m*M* NaHCO_3_, pH 7.62) at 39°C. Samples were transported (approximately 20 min) to the Animal Production Laboratory at the Public University of Navarre in thermos containers filled with water warmed to that same temperature.

After chilling for 24 h at 4°C, carcasses were weighed (cold carcass weight) and back fat thickness was measured using a calibre on both half-carcasses at a point located 4 cm from the spinal column laterally behind the last rib. The 10^th^ rib from the left carcass side was removed, weighed and stored at 4°C for analysis. Bone, muscle and adipose tissue (SC and intermuscular) were determined gravimetrically after dissection using the methodology of Colomer-Rocher et al. [12]. The *Longissimus dorsi* area at the 10^th^ rib and its content of IM fat was determined by image analysis technique [13]. Samples of SC and IM AT for FA analysis were taken from the 11^th^ to 13^th^ thoracic rib section from the left carcass side and frozen at -20°C. Samples for thiobarbituric acid reactive substances (TBARS) and sensory analysis were aged under vacuum in pouches of polyamide/polyethylene (120 μm and 1 cc/m^2^/24h O_2_ permeability, 3 cc/m^2^/24h CO_2_ permeability and 0.5 cc/m^2^/24h N_2_ permeability, measured at 5°C and 75% relative humidity; Vaeseen Schoemarket Ind. Spain) for 4 d postmortem in the dark and then frozen at -20°C until analysis.

### Adipocyte cellularity

The samples used to determine adipocyte size were digested with collagenase to dissolve the matrix of connective tissue surrounding the adipocytes [14]. The diameter of the adipocytes from each depot (200 approximately) was measured using an image analysis software (Image-Pro Plus 5.1) as described by Mendizabal et al. [15]. The number of adipocytes in the SC and IM AT were calculated based on the amount of adipose tissue, the mean adipocyte volume, the lipid content of the adipose tissue, and a lipid density value of 0.915 g/mL, assuming the cells are of spherical shape (lipid weight × chemical lipid content / 0.915 × mean adipocyte volume).

### Fatty acid composition

For FA determination, duplicate 1–2 g *Longissimus dorsi* muscle and 0.2–0.3 g SC AT was saponified in 6 ml of 5 *M* KOH in methanol/water (50:50, v/v) at 60°C for 60 min, and the extracted FA were methylated using 2 *M* trimethylsilyl-diazomethane in methanol:toluene (2:1, v/v) at 40°C for 20 min based on the method of Whittington et al. [16] as described in Urrutia et al. [5]. Analysis was performed by gas chromatography using a BPX-70 (SGE U.K. Ltd.) fused-silica capillary column (120 m × 0.22 mm i.d. × 0.2 μm film thickness). The fatty acid methyl esters were separated by gas chromatography (Agilent model 7890) using a flame ionization detector and hydrogen as the carrier gas. The oven temperature was initially set at 50°C, gradually ramped up to 240°C, and held there until the end of the cycle (15 min approximately). Fatty acid methyl esters were identified based on similar peak retention times using standards when available (Sigma Chemical Co. Ltd., Poole, UK). Fatty acids were quantified using tricosanoic acid methyl ester (C23:0), added prior to saponification, as an internal standard. The results were expressed as grams per 100 grams of total identified fatty acid methyl esters. Column response and linearity were checked using a mixture of fatty acids (C16:0, C18:0, C18:1 *n*-9, C18:2 *n*-6, relative to internal standard C23:0, Sigma Chemical Co. Ltd., Poole, UK). In these chromatographic conditions, separation of the isomers C18:1 *t-*10 and C18:1 *t-*11 was not possible in all samples, and thus, C18:1 *t-*10 + C18:1 *t-*11 was reported.

### Gene expression

Total RNA was isolated from 100 mg of SC AT using the RNasy Lipid Tissue Mini Kit (Qiagen, Hilden, Germany) and from 200 mg of *Longissimus dorsi* using the GenElute Mammalian total RNA Miniprep Kit (Sigma–Aldrich Química, Madrid, Spain) according to the manufacturers' instructions. Concentration and purity of the total RNA were calculated using a Nanodrop 2000 spectrophotometer (Thermo Scientific, Madrid, Spain). The RNA was treated with DNase to remove genomic DNA using RQ1 RNases-Free DNase (Promega Corporation, Madison, WI, USA) and single-stranded cDNA was synthesized from 1.5 μg of total RNA using M-MLV Reverse Transcriptase (Promega Corporation, Madison, USA) according to the manufacturer´s instructions.

Four reference genes, including *β-actin (ACTB)*, *cyclophilin (PPIA)*, *18S ribosomal RNA (18S RNA)* and *glyceraldehyde phosphate dehydrogenase (GAPDH)*, were analyzed by using the GeNorm program [17] to determine the suitable reference gene to express accurately the relative gene expression of the selected markers. The overall stability of the tested reference genes was measured by calculating the gene expression stability (M-value): *β-actin* showed the best M-value (0.16), below the 1.5 cut-off value specified by the GeNorm program. Adding additional reference genes did not decrease the M-value, indicating there was no increase in stability by using extra reference genes. Therefore, *β-actin* was selected as reference gene for normalization. *Acetyl-CoA carboxylase 1* (*ACACA****)***, *lipoprotein lipase* (*LPL*), *stearoyl-CoA desaturase (SCD)*, *peroxisome proliferator-activated receptor gamma (PPARG) and CAAT-enhancer binding protein alpha (CEBPA)*, *sterol regulatory element-binding factor 1* (*SREBF1*), *fatty acid desaturase 1* (*FADS1*), *fatty acid desaturase 2* (*FADS2*) and *fatty acid elongase 5* (*ELOVL5*) expression was quantified by real-time qPCR. Oligonucleotides for the real time qPCR (S1 Table) were designed using Primer3 Software (http://frodo.wi.mit.edu/primer3/). Relative transcript quantification of samples was performed using an ABI PRISM 7900 Sequence Detector (Applied Biosystems, Madrid, Spain). PCR products were cloned into pGEM-T Easy vector (Promega Corporation, Madison, USA) and chemically transformed into NEB 10-beta *E*. coli (New England Biolabs Inc., UK). qPCR efficiency was estimated by standard curve calculation using a 10-fold dilution series of plasmid DNA and confirmed using a 10-fold dilution series of pooled cDNA. Real-time qPCR reactions to compare variability among groups and standard curves were performed in a total volume of 25 μl containing 5 μl pooled cDNA template, 6 μl SYBR Premix Ex Taq (Takara, Japan), 0.6 μl forward and reverse primers (5 μM each), and 12.8 μl DNase/RNase free water. Real-time qPCR conditions were: 50°C/2 min, 95°C/10 min, 45 cycles of 95°C/15 s and 60°C/1 min, followed by amplicon dissociation (95°C/15 s, 60°C/15 s, 95°C/15 s). Dissociation curves were examined for the presence of a single PCR product. Efficiency of PCR amplification for each gene was calculated using the standard curve method (*E* = 10^−1/*slope*^) [18] and relative gene expression was calculated with the comparative, efficiency-corrected ΔΔC_T_ method [19], using *β-actin* for gene expression normalization.

### Meat quality and sensory analysis

Carcass color [20] of the *Lattisimus dorsi* muscle and SC AT (12^th^ rib) was measured at 24 h postmortem using a Minolta CM2002 spectrophotometer (D65 illuminant, 8-mm diameter aperture, 10° standard observer, 8° viewing angle; Minolta Inc., Osaka, Japan) and lightness (L*), redness (a*) and yellowness (b*) were recorded.

The pH value of the *Longissimus dorsi* (6^th^ and 12^th^ rib) was determined at 24 h postmortem with an Orion Research Potentiometer (Orion Research Inc., Barcelona, Spain) for solid samples. Thiobarbituric acid reactive substances (TBARS) were analysed in meat aged 4 d postmortem by the method of Tarladgis et al. [21]. Absorbancies at 532 nm, measured with a spectrophotometer (model UV-2101 PC; Shimadzu, Japan), were converted to milligrams of malonaldehyde per kilogram meat and reported as TBARS values.

The sensory evaluation of the meat aged 4 d postmortem was conducted by an 88-member consumer panel. Consumers were recruited through a specialized company by gender (46.6% male and 53.41% female) and age (18–30 years, 30–45 years, > 45 years) with the restriction that selected panelists had to be consumers of lamb meat. *Longissimus dorsi* meat samples were thawed at 4°C for 24 h and were evaluated after grilled (model BBC-842 Grill 230 V-2,000 W, Fagor Inc., Spain) at 200°C until reaching an internal temperature of 70°C. Immediately, the samples were cut into portions of 1.5 cm long, wrapped in an aluminium foil, and kept warm for 5 min maximum in a sand bath until sensory evaluation. There were 11 testing sessions (8 panelists per session). Panelists were offered three samples (one sample per treatment) in a random order and the 8 panelists evaluated the same combination of three samples. The only information given to each panelist prior to tasting was that the sample was of lamb meat. Each panelist was supplied with unsalted saltine crackers and mineral water for palate cleansing between samples. The consumers were asked to evaluate each sample based on odor, flavor, tenderness, juiciness and a global acceptability using an 8-point hedonic scale, which ranged from “dislike very much” to “like very much”. Panelists were also asked to record comments on atypical flavors.

### Statistical methods

Results were analysed statistically using one-way ANOVA (IBM SPSS Statistics 21.0) to determine the main effect of diet. Previously, adipose tissue weight and adipocyte number and diameter and sensory data were logarithmically (log_10_) transformed to satisfy the conditions of normality. Adipocyte size frequency distributions was studied by grouping according to adipocyte diameter (> 30 μm, 30–60 μm, 60–90 μm, 90–120 μm, and > 120 μm). The model used was:

$$y_{ij}=\mu +G_{i}+e_{ij}$$

where *y*_*ij*_ = growth and carcass parameters, adipocyte cellularity, FA composition, color, pH, TBARS and sensory variables; μ = mean value, *G*_*i*_ = fixed effect of group (*i* = 1: C; *i* = 2: L; *i* = 3: L-A); *e*_*ij*_ = random residual effect.

Differences among means were tested by Tukey´s HSD Test. Statistical significance was accepted at *P* < 0.05 and statistical trends at *P* < 0.10.

Differences in gene expression among diets were tested for statistical significance using REST algorithm (REST 2009; Relative Expression Software Tool, Version V2.0.13) [22].

## Results

### Growth and carcass parameters

Growth and carcass parameters are presented in Table 2. No differences in average daily gain (ADG) and slaughter age of lambs were observed in linseed fed lambs compared to the C group lambs (*P >* 0.05). By contrast, lambs fed L-A diet showed lower ADG, and therefore, greater slaughter age compared to C and L fed diets (*P <* 0.001). Feed intake of lambs in C, L and LA groups were 1,020, 1,002 and 790 g·lamb^-1^·day^-1^, respectively. Moreover, hot carcass weight and cold carcass weight of the three groups of lambs were not different (*P >* 0.05).

**Table 2:** Effects of addition of linseed or linseed and marine algae to the diet on growth and carcass parameters of Navarra breed lambs.

Item	Treatment^1^				
	C	L	L-A	SEM	*P*-value
Weaning weight, kg	16.2	16.3	16.4	0.32	0.955
Weaning age, d	52.6	55.6	57.1	1.54	0.137
ADG^2^, g/d	382^A^	350^A^	234^B^	15.7	<0.001
Slaughter age, d	80.6^B^	85.9^B^	99.0^A^	2.48	<0.001
Slaughter weight, kg	26.9	26.9	26.2	0.31	0.241
Hot carcass weight, kg	11.8	12.0	11.6	0.17	0.174
Cold carcass weight, kg	11.4	11.6	11.2	0.20	0.121

^A,B^Means with different uppercase superscripts within a row are different (*P <* 0.01).

^1^Treatments: C = control; L = 10% linseed; L-A = 5% linseed with 3.89% marine algae.

^2^Weaning to slaughter.

### Adipose tissue development

The composition of the 10^th^ rib and adipocyte cellularity of SC and IM AT are shown in Table 3. There were no significant differences in 10^th^ rib weight, back fat thickness, muscle and bone weights, SC and intermuscular fat content, and *Longissimus dorsi* area and fat content among three groups of lambs (*P >* 0.05).

**Table 3:** Effects of addition of linseed or linseed and marine algae to the diet on carcass traits of the 10^th^ rib, number and diameter of adipocytes in subcutaneous and intramuscular adipose tissue of Navarra breed lambs.

Item		Treatment^1^				
		C	L	L-A	SEM	*P*-value
Carcass characteristics of the 10^th^ rib						
10^th^ rib weight, g		74.2	77.2	75.9	2.85	0.753
Back fat thickness, mm		2.58	3.34	2.49	0.32	0.141
Muscle weight, g		36.0	34.8	33.7	1.43	0.527
Bone weight, g		18.8	18.4	18.2	1.16	0.928
Subcutaneous fat, g		11.5	13.5	12.5	1.04	0.419
Intermuscular fat, g		6.38	5.86	7.38	0.59	0.208
*Longissimus dorsi* area, cm^2^		14.9	15.4	14.7	0.44	0.555
*Longissimus dorsi* intramuscular fat, %		3.12	2.75	3.59	0.33	0.224
Adipocyte number, 10^6^						
Subcutaneous^2^		55.9	55.5	43.9	11.1	0.696
Intramuscular^3^		69.0	96.5	73.6	20.9	0.680
Adipocyte diameter						
Subcutaneous^2^	Mean, μm	55.2	64.5	67.6	3.01	0.057
	Relative frequency, %					
	< 30 μm	40.6^a^	33.6^a^^b^	27.8^b^	3.17	0.030
	30–60 μm	19.9^a^	13.0^b^	14.2^a^^b^	1.72	0.018
	60–90 μm	19.6	21.8	24.1	2.60	0.500
	90–120 μm	15.8^b^	23.6^a^^b^	28.2^a^	2.86	0.022
	> 120 μm	4.22	8.01	5.71	1.65	0.308
Intramuscular^3^	Mean, μm	32.7	31.4	32.6	1.44	0.777
	Relative frequency, %					
	< 30 μm	56.3	62.4	61.3	3.97	0.522
	30–60 μm	37.4	31.1	28.2	3.22	0.141
	> 60 μm	6.37	6.60	10.54	1.57	0.158

^a,b^Means with different lowercase supercripts within a row are different (*P <* 0.05).

^1^Treatments: C = control; L = 10% linseed; L-A = 5% linseed with 3.89% marine algae.

^2^Obtained from the subcutaneous fat at the 10^th^ rib.

^3^Obtained from the *Longissimus dorsi* at the 10^th^ rib.

**Fig 1:**
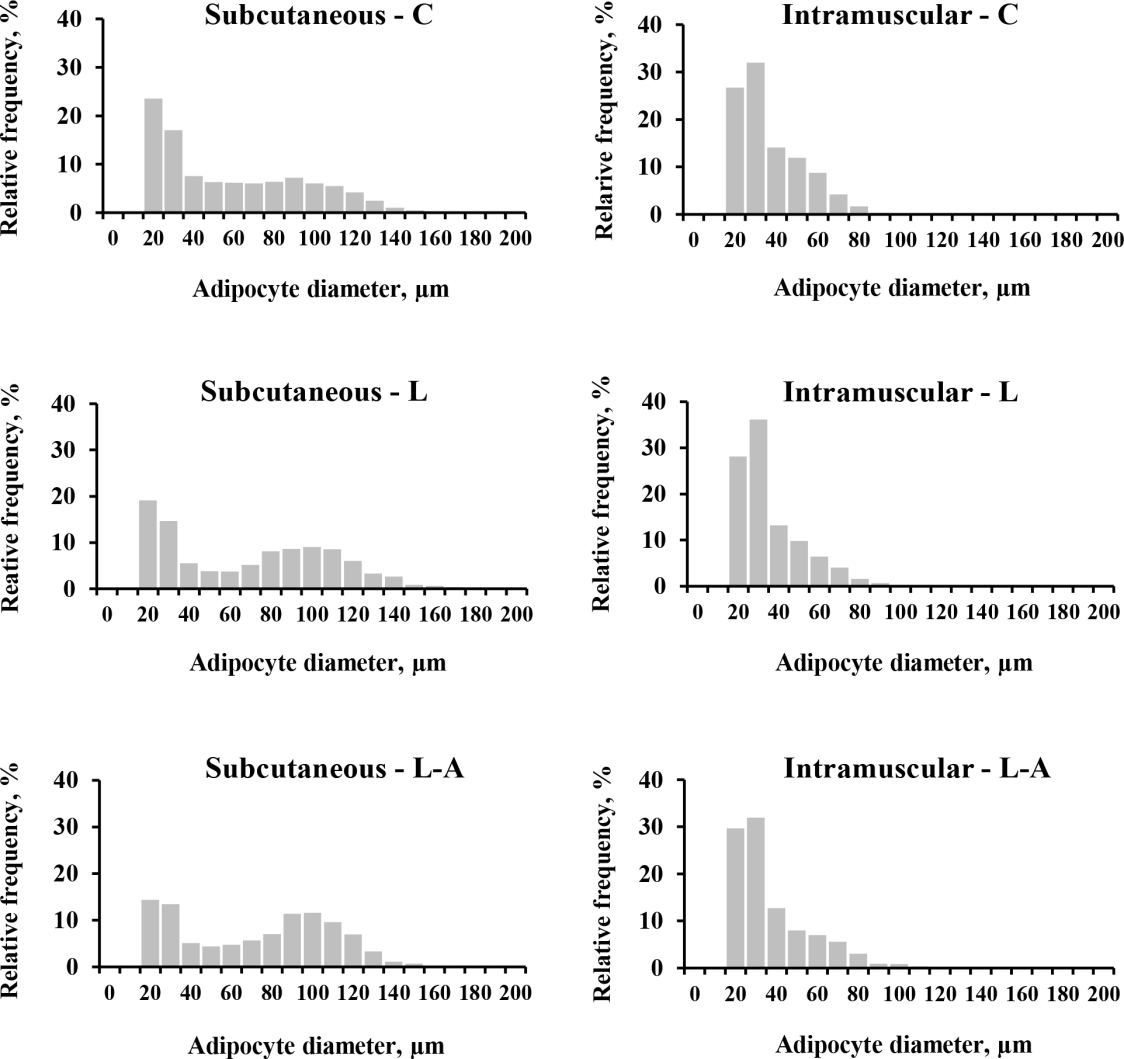
Effects of addition of linseed or linseed and algae to the diet on adipocyte size distribution of lambs. **A**, analysis of subcutaneous adipocyte size distribution of Navarra breed lambs. **B**, analysis of intramuscular adipocyte size distribution of Navarra breed lambs. C = control; L = 10% linseed; L-A = 5% linseed with 3.89% algae.

The number of adipocytes of IM and SC AT did not differ among three groups of lambs (*P >* 0.05). Mean IM adipocyte diameter was not different among dietary treatments (*P* > 0.05) whilst L and L-A group lambs adipocyte diameter showed a tendency to increase in SC AT compared to those fed control diet (*P* = 0.057). The adipocyte size distribution in SC AT revealed that the relative frequency of < 30 μm adipocyte class was lower in L-A group (*P <* 0.05) and 30–60 μm adipocyte class in L group lambs compared to C group lambs (*P <* 0.05). Moreover, the frequency of fat cells was significantly greater in 90–120 μm class in L-A group lambs (*P <* 0.05). Fig 1A illustrates the SC adipocyte diameter size bimodal distribution for the three groups of lambs, with peaks at 20–30 μm and 90–100 μm. On the other hand, Fig 1B shows IM adipocyte size asymmetrical distribution in lambs of the three dietary treatments. The largest proportion of the adipose cells were those with a 20–30 μm. The adipocyte size frequency distributions revealed no differences for < 30 μm, 30–60 μm and > 60 μm adipocyte classes among three groups of lambs in IM AT (*P >* 0.05).

### Fatty acid composition

Regarding some of the FA derived from ruminal biohydrogenation, the inclusion of linseed or linseed with algae in the diet increased the contents of C18:1 *t-*10 + C18:1 *t-*11 in both SC and IM AT compared to C group lambs (*P* < 0.001; Tables 4 and 5). Moreover, the content of C18:0 decreased in L-A group lambs compared to L group lambs in both SC and IM AT (*P <* 0.05). Additionally, C16:1 *c*-9 and oleic acid (C18:1 *c*-9) contents decreased in L and L-A group lambs in both AT (*P* ≤ 0.001). There were no differences in C18:2 *c*-9, *t*-11 (CLA) content in L group lambs in SC AT and L and L-A in IM AT compared to C group (*P >* 0.05), but decreased in L-A group lambs in SC AT compared to C and L groups (*P <* 0.001).

**Table 4:** Effects of addition of linseed or linseed and marine algae to the diet on fatty acid composition (g/100 g of total identified fatty acid methyl esters) in subcutaneous adipose tissue of Navarra breed lambs.

Item^2^	Treatment^1^				
	C	L	L-A	SEM	*P*-value
SFA					
C12:0	0.86^A^^a^	0.51^B^^b^	0.71^a^	0.05	<0.001
C14:0	6.31^A^	5.05^B^	6.35^A^	0.22	<0.001
C16:0	27.5^C^	26.0^B^	32.0^A^	0.38	<0.001
C18:0	11.9^a^^b^	12.8^A^	11.5^B^	0.30	0.028
C20:0	0.09^a^	0.08^b^	0.08^b^	0.00	0.030
Total SFA^3^	49.9^B^	49.9^B^	54.8^A^	0.80	<0.001
MUFA					
C16:1 *c-*9	1.86^A^	1.34^B^	1.37^B^	0.07	<0.001
C18:1 *t-*10 + C18:1 *t-*11	6.66^C^	13.41^A^	11.53^B^	0.50	<0.001
C18:1 *c-*9 (oleic acid)	28.4^A^	22.2^B^	21.4^B^	0.60	<0.001
C18:1 *c-*11	1.73^A^	1.13^B^	1.27^B^	0.05	<0.001
C20:1 *c-*11	0.04^b^	0.05^A^^a^	0.03^B^^b^	0.00	0.001
Total MUFA^3^	42.2^A^^a^	41.0^a^	38.1^B^^b^	0.70	0.001
C18:2 *c-*9, *t-*11 (CLA)	0.16^A^	0.15^A^	0.11^B^	0.01	<0.001
∑CLA^4^	0.40^B^	0.48^A^	0.31^C^	0.02	<0.001
*n*-3 PUFA					
C18:3 *n*-3 (ALA)	0.37^C^	1.92^A^	0.84^B^	0.05	<0.001
C20:5 *n*-3 (EPA)	0.07^B^	0.10^B^	0.21^A^	0.01	<0.001
C22:5 *n*-3 (DPA)	0.05^C^	0.08^B^	0.12^A^	0.01	<0.001
C22:6 *n*-3 (DHA)	0.02^B^	0.04^B^	0.58^A^	0.03	<0.001
Total *n*-3 PUFA	0.51^C^	2.14^A^	1.75^B^	0.05	<0.001
*n*-6 PUFA					
C18:2 *n*-6 *t-*9, *t-*12	0.10^C^	1.18^A^	0.50^B^	0.04	<0.001
C18:2 *n*-6 (LA)	3.93^A^	2.46^B^^c^	1.89^B^^d^	0.14	<0.001
C20:3 *n*-6	0.04^B^	0.05^B^	0.08^A^	0.05	<0.001
C20:4 *n*-6 (AA)	0.17^B^	0.15^B^	0.28^A^	0.01	<0.001
Total *n*-6 PUFA^3^	4.24^a^	3.84^a^^b^	3.25^a^	0.19	0.035
Total PUFA	4.57^B^	5.84^A^	4.51^B^	0.23	<0.001
*n*-6 PUFA/*n*-3 PUFA	8.32^A^	1.78^B^	2.82^C^	0.10	<0.001
PUFA/SFA	0.10^A^^B^	0.12^A^	0.08^B^	0.01	0.006

^a,b,c^Means with different lowercase supercripts within a row are different (*P <* 0.05).

^A,B,C^Means with different uppercase supercripts within a row are different (*P <* 0.01).

^1^Treatments: C = control; L = 10% linseed; L-A = 5% linseed with 3.89% marine algae.

^2^ALA = α-linolenic acid; EPA = eicosapentaenoic acid; DPA = docosapentaenoic acid; DHA = docosahexaenoic acid; LA = linoleic acid; AA = arachidonic acid.

^3^Total SFA, MUFA and *n*-6 PUFA is the sum of all SFA, MUFA and *n*-6 PUFA.

^4^∑CLA = C18:2 *c-*9, *t-*11 + C18:2 *t-*10, *c-*12 + C18:2 *c-*9, *c-*11 + C18:2 *t-*9, *t-*11.

**Table 5:** Effects of addition of linseed or linseed and marine algae to the diet on fatty acid composition (g/100 g of total identified fatty acid methyl esters) in intramuscular adipose tissue of Navarra breed lambs.

Item^2^	Treatment^1^				
	C	L	L-A	SEM	*P*-value
Total fatty acids (mg/100 g muscle)	3,125	2,943	3,158	196	0.726
SFA					
C12:0	0.57^A^	0.37^B^	0.38^B^	0.03	<0.001
C14:0	4.28^a^	3.67^b^	4.02^a^^b^	0.14	0.021
C16:0	27.3^C^	26.8^B^	31.5^A^	0.40	<0.001
C18:0	13.7^A^	13.5^A^	12.5^B^	0.20	0.001
C20:0	0.03	0.13	0.03	0.04	0.413
Total SFA^3^	48.9^b^	47.5^B^^b^	50.7^A^^a^	0.50	<0.001
MUFA					
C16:1 *c*9	1.64^A^^a^	1.42^b^	1.28^B^^b^	0.06	0.001
C18:1 *t-*10 + C18:1 *t-*11	2.88^B^	7.04^A^	7.93^A^	0.50	<0.001
C18:1 *c-*9 (oleic acid)	28.9^A^	24.9^B^^a^	21.3^B^^b^	0.80	<0.001
C18:1 *c-*11	1.84	1.68	1.63	0.10	0.369
C20:1 *c-*11	0.10^b^	0.40^a^	0.07^b^	0.06	0.040
Total MUFA^3^	37.1^A^^a^	36.3^a^	33.8^B^^b^	0.70	0.004
C18:2 *c-*9, *t-*11 (CLA)	0.12	0.11	0.16	0.02	0.450
∑ CLA^4^	0.26	0.30	0.29	0.02	0.652
*n*-3 PUFA					
C18:3 *n*-3 (ALA)	0.40^C^	1.84^A^	0.89^B^	0.09	<0.001
C20:5 *n*-3 (EPA)	0.19^B^	0.74^A^	1.01^A^	0.09	<0.001
C22:5 *n*-3 (DPA)	0.23^B^^b^	0.31^a^	0.32^A^^a^	0.02	0.002
C22:6 *n*-3 (DHA)	0.05^B^	0.08^B^	0.99^A^	0.02	<0.001
Total *n*-3 PUFA^3^	1.04^B^^b^	4.32^A^	3.21^a^	0.37	<0.001
*n*-6 PUFA					
C18:2 *n*-6 *t-*9, *t-*12	0.09^C^	0.45^A^	0.29^B^	0.03	<0.001
C18:2 *n*-6 (LA)	7.77	6.78	5.97	0.53	0.072
C18:3 *n*-6	0.08	0.08	0.07	0.04	0.295
C20:2 *n*-6	0.07^B^	0.07^B^	0.11^A^	0.01	<0.001
C20:3 *n*-6	0.19^a^	0.15^B^^b^	0.22^A^^a^	0.01	<0.001
C20:4 *n*-6 (AA)	2.71^b^	2.16^C^^c^	3.35^A^^a^	0.19	<0.001
C22:4 *n*-6	0.18^A^	0.13^B^	0.06^C^	0.01	<0.001
Total *n*-6 PUFA^3^	11.09	9.82	10.86	0.69	0.401
Total PUFA	12.2	14.2	13.9	0.60	0.051
*n*-6 PUFA/*n*-3 PUFA	10.46^A^	3.76^B^	4.44^B^	0.54	<0.001
PUFA/SFA	0.25^b^	0.30^a^	0.27^a^^b^	0.01	0.050

^a,b,c^Means with different lowercase supercripts within a row are different (*P <* 0.05).

^A,B,C^Means with different uppercase supercripts within a row are different (*P <* 0.01).

^1^Treatments: C = control; L = 10% linseed; L-A = 5% linseed with 3.89% marine algae.

^2^ALA = α-linolenic acid; EPA = eicosapentaenoic acid; DPA = docosapentaenoic acid; DHA = docosahexaenoic acid LA = linoleic acid; AA = arachidonic acid.

^3^Total SFA, MUFA, *n*-3 PUFA and *n*-6 PUFA is the sum of all SFA, MUFA, *n*-3 PUFA and *n*-6 PUFA.

^4^∑CLA = C18:2 *c-*9, *t-*11 + C18:2 *t-*10, *c-*12 + C18:2 *c-*9, *c-*11 + C18:2 *t-*9, *t-*11.

Both linseed and linseed and algae addition increased ALA levels in both SC (418.9% and 127.0% for L and L-A, respectively; *P <* 0.001) and IM AT (360.0% and 122.5% for L and L-A, respectively; *P <* 0.001). Moreover, the inclusion of linseed in the diet increased concentrations of ALA derivatives EPA in IM AT and DPA in both AT compared to C group (*P* < 0.01). However, tissue levels of DHA did not increase (*P >* 0.05). The proportions of EPA, DPA and also DHA increased in L-A group lambs compared to C in both AT (*P <* 0.01).

Linoleic acid (LA) content decreased in SC AT of L and L-A group lambs compared to C group (*P <* 0.001), but in the IM AT this FA content was similar among three groups of lambs (*P >* 0.05). Addition of linseed had no effect on the content of LA derivatives C20:3 *n*-6 and AA in SC AT and C18:3 *n*-6, C20:3 *n*-6 and AA of IM AT of lambs. On the contrary, the partial substitution of linseed with algae increased C20:3 *n*-6 and AA in L-A group lambs compared to L group lambs in both studied tissues (*P <* 0.001). Compared to C group lambs, L-A group lambs presented greater content of C20:3 *n*-6 and AA in the SC AT (*P <* 0.001), but these FA content were similar to C group in the IM AT.

The inclusion of either linseed or linseed in combination with algae resulted in a reduction of the *n*-6/*n*-3 ratio in the muscle of lambs (*P* < 0.001); however, only linseed addition reduced the *n*-6/*n*-3 ratio to a value lower than the recommended value of 4.0 due to the greater content of *n*-3 PUFA in the linseed diet. Additionally, feeding linseed increased PUFA/SFA ratio to a value close to the recommended 0.45 [23].

### Gene expression

The expression of *PPARG* and *CEBPA* in linseed or linseed with algae groups was greater than in control diet fed lambs in SC AT (*P <* 0.05; Table 6). By contrast, the expression of these transcription factors in IM AT of lambs was not affected by diet (*P >* 0.05). The expression of *SREBF1* was not affected by added linseed or linseed with algae in both SC and IM AT compared to C group lambs (*P* > 0.05). Furthermore, in the present work, the partial substitution of linseed with algae decreased the expression of *SREBF1* in the IM AT of L-A group lambs compared to L group lambs (the fold change value was 0.39 in L-A group relative to L group; *P* = 0.021).

**Table 6:** Effects of addition of linseed or linseed and marine algae to the diet on relative mRNA expression of lipid metabolism genes in subcutaneous and intramuscular adipose tissues of Navarra breed lambs.

Gene^1^	Tissue	Treatment^2^	FC^3^	Std. Error^4^	*P*-value	Result
*PPARG*	SC	L	7.42	2.99–17.29	0.012	UP
		L-A	10.6	1.57–50.19	0.032	UP
	IM	L	0.98	0.38–2.61	0.961	NR^5^
		L-A	0.87	0.27–2.39	0.771	NR
*CEBPA*	SC	L	1.94	1.29–2.80	0.024	UP
		L-A	8.13	1.48–23.80	0.040	UP
	IM	L	0.54	0.27–1.15	0.119	NR
		L-A	0.54	0.27–1.15	0.099	NR
*SREBF1*	SC	L	1.01	0.60–1.92	0.977	NR
		L-A	1.70	0.18–6.12	0.518	NR
	IM	L	1.12	0.43–3.11	0.813	NR
		L-A	0.44	0.14–1.18	0.110	NR
*SCD*	SC	L	0.83	0.49–1.43	0.213	NR
		L-A	0.51	0.37–0.88	0.003	DOWN
	IM	C/L	0.07	0.04–0.13	<0.001	DOWN
		L-A	0.53	0.29–0.95	<0.001	DOWN
*ACACA*	SC	L	0.74	0.53–1.25	0.044	DOWN
		L-A	0.09	0.05–0.13	<0.001	DOWN
	IM	L	0.61	0.37–0.98	<0.001	DOWN
		L-A	0.24	0.15–0.41	<0.001	DOWN
*LPL*	SC	L	1.18	0.73–1.77	0.169	NR
		L-A	0.52	0.34–0.78	0.002	DOWN
	IM	L	1.83	1.09–3.16	0.095	NR
		L-A	0.41	0.23–0.73	0.032	DOWN
*FASD1*	SC	L	2.13	0.49–16.33	0.609	NR
		L-A	0.26	0.08–0.92	0.086	NR
	IM	L	0.11	0.04–0.30	0.001	DOWN
		L-A	0.11	0.04–0.32	0.001	DOWN
*FASD2*	SC	L	3.15	0.48–14.22	0.236	NR
		L-A	0.42	0.14–1.54	0.293	NR
	IM	L	0.31	0.18–0.52	<0.001	DOWN
		L-A	0.13	0.06–0.22	<0.001	DOWN
*ELOVL5*^*6*^	SC	L	3.50	1.69–8.08	0.101	NR
		L-A	0.30	0.16–0.59	0.203	NR

^1^*PPARG = peroxisome proliferator-activated receptor gamma; CEBPA = CAAT-enhancer binding protein alpha; SREBF1* = *sterol regulatory element-binding factor 1*; *SCD* = *stearoyl-CoA desaturase*; *ACACA* = *acetyl-CoA carboxylase 1; LPL* = *lipoprotein lipase*; *FADS1* = *fatty acid desaturase 1*; *FADS2* = *fatty acid desaturase 2*; *ELOVL5* = *fatty acid elongase 5*.

^2^Treatments: L = 10% linseed; L-A = 5% linseed with 3.89% marine algae.

^3^FC = Fold change. The values reported represent the fold change relative to control group that has the value of 1.

^4^Std. Error. A range for Standard Error calculated by REST software.

^5^NR = not regulated.

^6^The relative gene expression of *ELOVL5* in IM AT was not detected.

There were no differences in the abundance *SCD* mRNA in linseed fed lambs in SC AT whereas feeding linseed with algae decreased the expression of *SCD* (*P* < 0.01). In the IM AT, the expression of *SCD* was downregulated in both L and L-A group lambs (*P* < 0.001). The expression of *ACACA* gene was significantly reduced by adding linseed and more intensively by linseed with algae in both SC (*P* < 0.05) and IM AT (*P* < 0.001). Furthermore, *ACACA* expression decreased in L-A group lambs compared to L group (*P ≤* 0.01). No differences in *LPL* gene expression (*P >* 0.05) were observed in linseed fed lambs, whereas linseed with algae-enriched diets decreased *LPL* mRNA levels in both SC (*P <* 0.01) and IM (*P* < 0.05) AT of lambs compared to C group lambs.

Adding either linseed or linseed with algae to the diet did not affect the expression of *FADS1*, *FADS2* and *ELOVL5* in the SC AT (*P* > 0.05). Conversely, in the IM AT, the expression of *FADS1* and *FADS2* genes in L and L-A group lambs was significantly downregulated (*P* < 0.001). Moreover, *FADS2* expression decreased in L-A group lambs compared to L group (*P* < 0.01).

### Meat quality and sensory analysis

Carcass pH did not differ among different treatments (*P >* 0.05; Table 7). Additionally, TBARS concentration was significantly greater in *Longissimus dorsi* samples from lambs fed linseed with algae compared to samples from lambs fed the C diet (*P <* 0.05). Regarding carcass color, the samples from SC AT of linseed fed lambs presented an increase in L* (ligthtness) and b* (yellowness) values (*P <* 0.01) and the SC AT of lambs fed linseed with algae showed a greater L* value and lower a* value than lambs fed the control diet (*P <* 0.001). In muscle samples, there were no differences in a* value (redness) among three dietary groups (*P >* 0.05). The b* value of L and L-A groups meat increased compared to C group meat and also L* value in L-A group meat (*P <* 0.001).

**Table 7:** Effects of addition of linseed or linseed and marine algae to the diet on meat quality traits of Navarra breed lambs.

Item	Treatment^1^				
	C	L	L-A	SEM	*P*-value
pH					
*Longissimus dorsi* at the 6^th^ rib	5.80	5.77	5.81	0.04	0.401
*Longissimus dorsi* at the 12^th^ rib	5.85	5.85	5.92	0.03	0.818
Carcass color^2^					
Subcutaneous adipose tissue^3^					
L*	64.4^B^	66.8^A^	67.6^A^	0.5	<0.001
a*	4.14^A^	4.26^A^	2.07^B^	0.21	<0.001
b*	10.5^b^	12.1^a^	10.7^b^	0.4	0.006
*Latissimus dorsi* muscle					
L*	52.6^B^	53.0^B^	56.9^A^	0.4	<0.001
a*	14.9	15.1	14.1	0.3	0.137
b*	13.1^B^	15.4^A^	16.5^A^	0.4	<0.001
*Longissimus dorsi* TBARS^4^	0.41^b^	0.53^a^^b^	0.74^a^	0.08	0.017

^a,b^Means with different lowercase supercripts within a row are different (*P <* 0.05).

^A,B^Means with different uppercase supercripts within a row are different (*P <* 0.01).

^1^Treatments: C = control; L = 10% linseed; L-A = 5% linseed with 3.89% marine algae.

^2^Objective color measurements; L*, a*, and b*, represents lightness, redness, and yellowness, respectively.

^3^Obtained from the SC AT at the 12^th^ rib.

^4^TBARS = thiobarbituric acid reactive substance (mg of malonaldehyde per kg of meat).

Sensory attributes of meat (odor, flavor, tenderness, juiciness and overall acceptability) did not differ in L group meat compared to C group (Table 8). Conversely, L-A group meat presented reduced ratings for odor, flavor and overall acceptability compared to C and L groups (*P <* 0.01). Furthermore, off-flavors were detected by 48.9% of the panelists, from which 13.6% of the consumers identified this off-flavor as “fishy flavor”.

**Table 8:** Effects of addition of linseed or linseed and marine algae to the diet on sensory evaluation of *longissimus dorsi* muscle.

Item^2^	Treatment^1^				
	C	L	L-A	SEM	*P*-value
Odor	6.14^A^	5.84^A^	5.18^B^	0.13	<0.001
Flavor	5.98^A^	5.82^A^	4.94^B^	0.14	<0.001
Tenderness	5.99	6.11	5.67	0.14	0.075
Juiciness	5.91^a^^b^	6.06^a^	5.56^b^	0.14	0.034
Overall acceptability	6.07^A^	5.93^A^	5.14^B^	0.13	0.001

^a,b^Means with different lowercase supercripts within a row are different (*P <* 0.05).

^A,B^Means with different uppercase supercripts within a row are different (*P <* 0.01).

^1^Treatments: C = control; L = 10% linseed; L-A = 5% linseed with 3.89% marine algae.

^2^Scored using an 8-point hedonic scale: 0 = “dislike very much”; 8 = “like very much”.

## Discussion

The absence of differences in ADG of L group lambs compared to the C group lambs are in agreement with previous results for Navarra breed lambs fed a diet with the same level of linseed [5]. The results are consistent with those published by Wachira et al. [24] and de la Fuente-Vázquez et al. [25], who reported no differences in these parameters in lambs fed 10.5% and 12.5% of linseed, respectively.

By contrast, the inclusion of algae in combination with linseed (3.89% and 5% DM, respectively) in the diet reduced ADG and hence the slaughter age of lambs increased. The reduction of ADG could be attributed to decreased feed intake, which might be caused by reduced palatability of diets with algae. Moreover, as indicated by works studying the inclusion of fish oil, ADG decrease might also be related to a decrease in microbial growth in the rumen [26] and fibre degradation [27]. In previous studies in which the percentage of algae was lower compared with the levels used in the present study, e.g. 1.92% DM of algae addition [28] and up to 3% DM [29], there was not a reduction in feed intake of lambs. Thus, from these results it could be drawn that levels higher than 3% DM of algae in the diet of lambs could cause a decrease in feed ingestion. Nevertheless, further research is necessary to understand effects of algae inclusion on DMI. Pair-feeding studies may be an useful tool to exclude effects in the animals by differences of feed intake.

The similarity among treatments in carcass characteristics, such as tisular composition of the 10^th^ rib or back fat thickness, is consistent with the results of other authors. De la Fuente-Vázquez et al. [25] did not observe differences in dorsal fat thickness, fat score and the percentage of fat in muscle in lambs fed algae (*Isochrysis* sp.; 4%) and linseed (10.7%) diet. Similarly, Atti et al. [30] fed up to 10% fish meal to lambs and observed no differences in carcass characteristics.

The adipocyte size frequency distributions of SC and IM adipocytes were analysed to gain knowledge of hypertrophy and hyperplasia. Bimodality in frequency histograms of adipose cell diameters may indicate the simultaneous occurrence of both hyperplasia, represented by the population of small cells, and the hypertrophy, represented by the large adipocyte population, during the early development stage of the SC AT in C, L and L-A groups of lambs. These results are in agreement with previous reports showing that the period around 4 months of age is a stage of an important hyperplasic component in the development of the SC AT, followed by a more rapid hypertrophy of these small adipocytes [31,32]. With reference to dietary treatment effect, the adipocyte size frequency distributions of SC AT revealed significantly greater relative frequency of 90–120 μm adipocytes from L-A lambs compared to C group lambs. Furthermore, the frequency of this adipocyte class for L group was 23.64% *vs*. 15.76% for C group lambs, although the difference was not statistically significant. When mean adipocyte diameter was compared among three lamb groups, there was also a tendency for adipocyte diameter to increase in L and L-A group lambs in SC AT. These results suggest that the hypertrophy process seemed to be stimulated in L and L-A treatments. Furthermore, the high frequency of 90–120 μm adipocytes observed in lambs fed linseed in combination with algae could be related to the fact that these lambs were slaughtered at significantly greater age than C and L lambs to reach the final slaughter weight. On the other hand, the lower frequency of small adipocytes in L (30–60 μm diameter class) and L-A groups (< 30 μm diameter class) may be due to the conversion of small adipocytes into large adipocytes probably caused by the higher fat content of L and L-A diets compared to C diet. In this sense, Boque et al. [33] reported that rats fed with high-fat diet developed adipocyte hypertrophy in the SC AT without changes in adipocyte number. Fig 1B shows the unimodal IM adipocyte size distribution in the three dietary treatments. In all the groups, the largest proportion of adipocyte cells were those with a 20–30 μm diameter. This is consistent with the fact that the IM AT is a late-developing fat depot compared with SC AT [34,35]. Lack of differences among all adipocyte classes of IM AT were consistent with the similar mean adipocyte diameter and the similar percentage of IM fat among three dietary groups.

A relationship between adipogenic/lipogenic genes and adipocyte cellularity may exist [36,37]. The transcription factors *PPARG* and *CEBPA* are central regulators of adipogenesis, through an induction of the expression of many downstream target genes involved in lipid metabolism [38,3]. *PPARG* is expressed during the late stage of adipocyte differentiation and remains abundantly expressed in differentiated adipocytes [39]. In the SC AT, greater expression of *PPARG* and *CEBPA* in L and L-A group lambs agree with the results reported by Ebrahimi et al. [40], who observed that supplementing linseed oil (0.4 or 1.3% DM) increased the *PPARG* expression in SC AT of growing goats, and by Kronberg et al. [41], who demonstrated a significant increase in *PPARG* mRNA level as a result of an increased *n*-3 FA levels in bovine muscle. Furthermore, Nakamura et al. [39] stated that when PUFA are included in the diet, the profile of *PPARG* targets indicates that the main effect of *PPARG* on glucose metabolism is to increase the activity of G3PDH involved in triglyceride synthesis rather than the induction of *de novo* lipogenesis. Thus, the increase in *PPARG* and *CEBPA* expression of SC AT in the present study may be consistent with the trend towards greater adipocyte diameter of L and L-A group lambs. By contrast, in the IM AT, there were no differences among treatment groups in the expression of *PPARG* and *CEBPA*, which concurs with the lack of differences in adipocyte diameter and adipocyte size frequency distributions among three groups of lambs. These results suggest the differences in the regulation of *PPARG* and *CEBPA* expression by dietary PUFA between both AT could be related to metabolic specificities of each depot [35,42].

The transcription factor *SREBF1* is involved in adipocyte differentiation and cholesterol and FA synthesis, activating genes required for lipid metabolism [43,44]. In this study, the expression of the transcription factor *SREBF1* was not affected by adding linseed or linseed with algae in both SC and IM AT compared to C group lambs. These results are in agreement with the results of Dervishi et al. [45] who reported the *SREBF1* mRNA level in IM AT of lambs was not affected by dietary PUFA (alfalfa grazing). Furthermore, in the present work, the partial substitution of linseed with algae decreased the expression of *SREBF1* in the muscle of L-A group lambs compared to L group lambs. This is consistent with the results of Waters et al. [46] who observed *SREBF1* decreased in bovine muscle when animals were fed a high LCPUFA diet (soybean and fish oil).

It is well known that rumen microbial population transforms unsaturated 18-carbon FA such as ALA and LA through ruminal biohydrogenation; however, it is not well documented if the same processes occur for LCPUFA, such as DHA [47]. *Trans* FA are produced as intermediates during the ruminal biohydrogenation of ALA and LA yielding stearic acid (C18:0) as the final product [48]. On the other hand, it has also been observed PUFA addition modifies the biohydrogenation of PUFA resulting in an accumulation of intermediates, including C18:1 *t-*10 or C18:1 *t-*11 [49]. The results of the present study could suggest a shift in the ruminal biohydrogenation caused by dietary PUFA, which resulted in an increase in the contents of C18:1 *t-*10 + C18:1 *t-*11 in both SC and IM AT in lambs fed either linseed or linseed with algae compared to C group lambs. Moreover, the partial substitution of linseed with algae reduced C18:0 content in both SC and IM AT compared to L group, suggesting algae exerted more inhibitory effects than linseed in the final biohydrogenation step in the rumen. The inhibitory effect of algae on PUFA biohydrogenation was also reported by Boeckaert et al. [50] and Meale et al. [29] in dairy cows and in growing lambs, respectively. Lourenço et al. [51] stated microalgae or fish oil, both rich in EPA and DHA, directly inhibit biohydrogenation and also the growth of ruminal bacteria.

Unlike the ruminant, dietary fatty acids in the non-ruminant are absorbed unchanged before incorporation into the tissue lipids and therefore, dietary lipid sources have a direct and generally predictable effect of fatty acid composition of non-ruminant products [52]. In pigs, Huang et al. [53] fed animals with 10% of linseed 30 d before slaughter and observed higher ALA contents in *longissimus dorsi* muscle (2.46 g/100 of total FA) and SC fat (4.54 of g/100 g of total FA) compared to the results of this study (1.92 g/100 g of total FA in IM AT and 1.84 g/100 g of total FA in SC AT). Similarly, Juárez et al. [54] observed higher percentages of ALA in back fat (5.52 g/100 g of total FA) of pigs fed 10% of linseed for 4 weeks. Moreover, Mourot et al. [55] also observed a higher enrichment in ALA and DHA in tissues than the present study when linseed, linseed-microalgae mixture or microalgae was included in pig’s diets and Sardi et al. [56] reported DHA values of 0.23 g/100 g of total FA in *longissimus dorsi* muscle and 0.13 g/100 g of total FA in SC fat in pigs fed a diet containing lower percentage of marine algae (0.5%) than the inclusion level in the present work.

In this study, although the content of C18:1 *t-*10 + C18:1 *t-*11 increased in both SC and IM AT, the beneficial fatty acid C18:2 *c-*9, *t-*11 (CLA) synthetized from C18:1 *t-*11 was unaffected in L group lambs in SC AT and L and L-A in IM AT; even it decreased in L-A in SC AT. Therefore, a high dietary ALA does not necessarily lead to an increased C18:2 *c-*9, *t-*11 (CLA) content in AT, even if it increases C18:1 *t-*11 in both SC and IM AT [24].

Δ9 desaturase (codified by *SCD* gene) plays an important role in conversion of C18:1 *t-*11 into C18:2 *c-*9, *t-*11 (CLA) and it is also responsible for converting SFA to MUFA, primarily stearic acid (C18:0) into oleic acid (C18:1 *c-*9). It is plausible that as a result of the inhibition of *SCD* by dietary PUFA the content of C18:2 *c-*9, *t-*11 (CLA) did not increase in L and even decreased in L-A group in SC AT, despite the fact that its precursor C18:1 *t-*11 was available. Also, the content of C16:1 *c*-9 and oleic acid, synthesized by Δ9 desaturase, decreased in SC and IM AT compared to C group.

The expression of *ACACA* gene, which encodes the enzyme responsible for *de novo* FA synthesis (acetyl-CoA carboxylase), was significantly reduced by adding linseed and more intensively by linseed with algae in both SC and IM AT. These results suggest that adding PUFA may decrease the *de novo* FA synthesis, at least at the transcriptional level. These results concur with those published by Hiller et al. [8], who reported *ACACA* gene expression was negatively affected by *n*-3 FA (grass-silage based diet) of steers in both SC AT and IM AT, and by Dervishi et al. [57] who found alfalfa grazing lambs had lower levels of *ACACA* and consequently lower levels of FA synthetized *de novo*. It is noteworthy that acetyl-CoA carboxylase determines the rate of FA synthesis and, among other regulatory mechanisms, it is regulated not only at the transcriptional level but also allosterically and by phosphorylation, thereby permitting the rate of FA synthesis to fluctuate in response to physiological and development conditions [58]. However, the data of the present study revealed that dietary PUFA led to transcriptional regulation of *ACACA* gene.

Lipoprotein lipase, which is encoded by *LPL* gene, is a rate-limiting enzyme involved in the uptake of triacylglyceride-derived FA. Adding linseed to lamb diets did not affect *LPL* gene expression, whereas linseed with algae-enriched diets caused a decrease in *LPL* mRNA levels in both SC and IM AT compared to C diet. This fact suggests that the inclusion in the diet of algae led to a transcriptional regulation of FA uptake, probably due to the high proportion of more unsaturated and longer chain FA (EPA and DHA). Duckett et al. [59] allowed steers to graze pasture rich in ALA and observed *LPL* mRNA level was not affected whilst Corazzin et al. [7] fed 8% linseed to bulls and observed a reduction in *LPL* expression in SC AT. Thus, the results of the present study and of papers published by other researchers suggest PUFA levels in the diet and the degree of unsaturation of FA could affect the expression of this gene in a different manner.

As expected, the inclusion of linseed increased ALA levels in both SC and IM AT and the proportion of ALA in L-A treatment lambs was intermediate between L and C groups, reflecting the dietary concentration of this FA. In respect to the *n*-3 LCPUFA, the synthesis of *n*-3 LCPUFA (C18:4 *n*-3, C20:4 *n*-3, EPA, docosapentaenoic acid [DPA, C22:5 *n*-3], C24:5 *n*-3, C24:6 *n*-3 and DHA) from ALA is performed in the endoplasmic reticulum through a series of alternating desaturations and elongations [60]. When linseed was included in the diet of lambs there were evidences that elongation and desaturation of ALA had occurred, with increased concentrations of EPA in IM AT and DPA in both AT compared to C group. However, tissue levels of DHA did not increase, which could be the result of the limited conversion of ALA to their LCPUFA products [29,61,62]. Conversely, the partial substitution of linseed with algae increased the proportions of EPA, DPA and also DHA in both AT, indicating the deposition of DHA can more easily be obtained by including algae in the diet rather than by desaturation and elongation of ALA.

Linoleic acid (LA, C18:2 *n*-6) is the precursor of the *n*-6 LCPUFA series formed by elongation and desaturation (C18:3 *n*-6, C20:3 *n*-6, arachidonic acid [AA, C20:4 *n*-6], C22:4 *n*-6, C24:4 *n*-6, C24:5 *n*-6 and C22:5 *n*-6) [63]. The decreased LA content in SC AT of L and L-A group lambs could be the result of the lower dietary LA and its ruminal biohydrogenation. On the other hand, the results regarding the effect of dietary PUFA on the contents of *n*-6 LCPUFA are not clear. Berthelot et al. [64] reported the proportions of C18:3 *n*-6, C20:3 *n*-6, AA, C22:4 *n*-6 decreased in the muscle of lambs fed high levels of ALA (linseed diet). Wachira et al. [24] also observed linseed-rich diet lowered both C20:3 *n*-6 and AA in muscle, but diets rich in fish oil (lower content of ALA in the diet) increased C20:3 *n*-6. Moreover, Ashes et al. [65] indicated that fish oil did not affect AA in muscle phospholipids, which the authors attributed to adequate Δ6 desaturase activity ensuring the conversion of LA to AA. Therefore, the results of the present study and those of other studies suggest the high levels of ALA in linseed diet and hence, high ALA/LA ratio, decreased the conversion of LA to *n*-6 LCPUFA, as the enzymes have a preference for *n*-3 PUFA [60]. In contrast, the FA sources that led to lower ALA/LA ratio in the diet, such as algae or fish oil, did not inhibit the LA elongation.

Δ5 desaturase, Δ6 desaturase (codified by the genes *FADS1* and *FADS2*, respectively) and elongase 5 (*ELOVL5*) enzymes are involved in the synthesis of *n*-6 and *n*-3 LCPUFA. The results showed that dietary PUFA affected the expression of these genes in a tissue specific-manner. Herdmann et al. [66] and Hiller et al. [8] also reported dietary *n*-3 PUFA decreased Δ6 desaturase protein expression in IM AT of bulls, but not in SC AT. The reason for a tissue-specific response of genes involved in FA polyunsaturation is not clear. Phospholipids are a major component of membrane lipids and hence are found in higher level in the IM lipid fraction [52]. Additionally, Raes et al. [62] reported a large change in the FA profile of the cell membranes could alter their properties and other physiological functions. Thus, the PUFA proportions of the phospholipids have to be strictly regulated, especially in the IM AT which was characterized by smaller adipocyte size than the SC AT, as it was reported by Schoonmaker et al. [67] and observed in the present study (Fig 1A and 1B). On the other hand, the LCPUFA added to L-A diet led to a more pronounced downregulation of *FADS2*, a key rate-limiting gene in the synthesis of LCPUFA, in the IM AT compared to L group. These results indicate, once more, that the type of dietary FA is one of the factors influencing the regulation of the endogenous synthesis of FA.

The observed increase in EPA, DPA and DHA was not consistent with decreased expression of genes involved in LCPUFA formation in IM AT, which is in agreement with previous studies in lambs [5] and steers [8]. The regulation of FA synthesis enzymes may occur through several potential mechanisms including transcriptional, posttranscriptional, and posttranslational processes [68]. Hence, it seems plausible that the mRNA levels of these genes are not necessarily correlated with their protein concentrations or enzyme activities, and the transcriptional regulation of the LCPUFA synthesis could be a more long-term regulation.

Overall, compared with the linseed diet, feeding linseed in combination with algae resulted more effective in reducing the expression of lipogenic genes, such as *ACACA* or *LPL*, in both SC and IM AT in addition to *FADS2* gene mentioned above. Nevertheless, the response to the linseed with algae diet of adipogenic transcription factors was similar to the effect observed in lambs fed the linseed diet.

With reference to meat quality, carcass pH did not differ among different treatments. This result confirms the lack of significant differences previously observed in meat pH due to linseed addition in lambs [69]. Regarding carcass color, there was a slight effect of feeding linseed or linseed with algae on *Latissimus dorsi* and SC AT color parameters. Subcutaneous fat from L-A treatment showed less red color than those of C and L treatments. This may be due to the lipid oxidation of this depot, as Yin and Faustman [70] reported that the oxidation of fat is related to the formation of precursors of oxymyoglobin oxidation and thus to the formation of metamyoglobin and the decrease in a*. However, in muscle samples the redness value was not affected by dietary treatments. The more yellow fat in L diets fed lambs and muscle in L and L-A could be related to the presence of carotenes and xanthophylls in linseed and marine products. The lipid oxidation results showed significantly greater TBARS concentration in IM AT from lambs fed linseed with algae compared to samples from lambs fed the C diet. The greater proportion of the longer chain PUFA (EPA, DPA and DHA) in IM AT of L-A group lambs could be the reason for the lower oxidative stability. By contrast, although linseed diet was high in PUFA, it did not affect IM AT oxidative stability. Kouba and Mourot [71] reported linseed presents a high content of ALA, which is susceptible to oxidation, even if it is less than the susceptibility of EPA and DHA. In accordance with the results of the present study, Nute et al. [72] observed the use of fish oil alone or mixed with marine algae containing diets showed greater oxidation and lower color stability in lamb meat. Vatansever et al. [73] reported when linseed oil, fish oil or combination of the two were fed to beef animals, the fish oil diet produced meat with a reduced vitamin E concentration and greater TBARS than other diets.

The most important criterion for consumer acceptability of *n*-3 PUFA-supplemented foods is sensory quality, which seems to be inversely related to the *n*-3 PUFA content [72]. The inclusion in the diet of linseed did not affect the organoleptic quality of the meat, which is consistent with the lack of effect of linseed on lipid oxidation. Nevertheless, the increase in the proportions of the LCPUFA in IM AT and a reduction in the ratings for odor and flavor acceptability was observed in meat from lambs fed linseed with algae. Off-flavors were detected, identified by consumers as “fishy flavor”, and resulted in its lower overall acceptability in L-A group lambs compared to C and L groups, which may be associated with the greater TBARS in IM AT of L-A group lambs. The proportion of DHA in L-A group lambs was higher than C and L group lambs and hence, the greatest number of double bonds were available for oxidation. These results are consistent with the finding of Nute et al. [72] who reported that linseed oil fed lambs were rated significantly better for overall acceptability than protected lipid supplement and marine algae fed lambs. Regarding the DHA level in meat and its effects on off-flavor detection, previous studies reported unacceptably high flavor and odor associated with muscle DHA levels of 5.35 g/100 g of total FA in lambs [72] and 1.5 g/100 g in pigs [74]. The result of this study showed that off-flavors were detected at lower DHA levels (0.99 g/100 g). However, it is difficult to value the limiting point at which meat fishy flavor could be detected due to an increase in DHA and future studies must be performed to define the optimum level and duration of n-3 PUFA supplementation in order to limit the risk of lipid oxidation and adverse effects on product quality.

## Conclusions

The results indicate that feeding linseed did not affect growth parameters of Navarra breed lambs; nevertheless, the partial substitution of linseed with marine algae reduced ADG and increased slaughter age of lambs. Dietary linseed or linseed and algae decreased the expression of *ACACA* and *SCD* in both SC and IM AT and *FADS1*, *FADS2* and *ELOVL5* in the IM AT, suggesting a regulation in *the novo* FA synthesis and a shift in monounsturared FA formation.

With reference to adipose tissue development, differences in cellularity as well as in expression of genes related to differentiation and lipogenesis and LCPUFA synthesis, indicate that SC and IM adipose tissues may possess distinct regulation, which results in different fat accretion and varied fatty acid distribution. Supplementing diets with linseed or linseed in combination with algae led to an enrichment of C18:3 *n*-3 and reduced *n*-6/*n*-3 ratio in both SC and IM adipose tissues. Algae addition seemed to be more effective than linseed addition in increasing the physiologically important EPA and DHA content in the studied tissues, but it had adverse effects on meat quality, as the meat from lambs fed linseed with algae presented greater lipid oxidation and reduced ratings for odor, flavor and overall acceptability. The content of ALA, LA and LCPUFA in the diet of lambs is an important factor in the nutritional regulation of lipid metabolism, especially in the LCPUFA synthesis, and further work is needed to elucidate the molecular and biochemical mechanisms controlling the synthesis and deposition of these fatty acids.

## Supporting Information

S1 Table Primes used for the quantification of the mRNA expression by real-time PCR. ^1^*ACACA = acetyl-CoA carboxylase 1; LPL = lipoprotein lipase; SCD = stearoyl-CoA desaturase*; *PPARG = peroxisome proliferator-activated receptor gamma 2*; *CEBPA = CAAT-enhancer binding protein alpha*; *SREBF1 = sterol regulatory element binding factor 1*; *FADS1 = fatty acid desaturase 1; FADS2 = fatty acid desaturase 2; ELOVL5 = fatty acid elongase 5; β-actin = beta actin*. ^2^*R*^2^ stands for the multiple coefficient of determination of the standard curve. ^3^Efficiency (E) is calculated as [10^−1/*slope*^].
(DOCX)
