# Supplementary material for: Effects of Addition of Linseed and Marine Algae to the Diet on Adipose Tissue Development, Fatty Acid Profile, Lipogenic Gene Expression, and Meat Quality in Lambs
Source: PLoS One. 2016 Jun 2;11(6):e0156765. doi: 10.1371/journal.pone.0156765 (PMC4890764; doi:10.1371/journal.pone.0156765)
Supplement: S1 Table — 1ACACA = acetyl-CoA carboxylase 1; LPL = lipoprotein lipase; SCD = stearoyl-CoA desaturase; PPARG = peroxisome proliferator-activated receptor gamma 2; CEBPA = CAAT-enhancer binding protein alpha; SREBF1 = sterol regulatory element binding factor 1; FADS1 = fatty acid desaturase 1; FADS2 = fatty acid desaturase 2; ELOVL5 = fatty acid elongase 5; β-actin = beta actin. 2R2 stands for the multiple coefficient of determination of the standard curve. 3Efficiency (E) is calculated as [10−1/slope]. (DOCX) [file pone.0156765.s001.docx]

**S1 Table. Primes used for the quantification of the mRNA expression by real-time PCR.**

| Gene^1^ | Accession  number | Primer sequence,  5’-3’ | Size, bp | (*R*^2^)^2^ | (E)^3^ |
| --- | --- | --- | --- | --- | --- |
| *ACACA* | X80045 | F: ATGACCTTAACAGCCGCAGAGT  R: CTATTACACAGCCCGGGTCAAG | 82 | 0.97 | 1.97 |
| *LPL* | NM_001009394 | F: TCATCGTGGTGGACTGGCT  R: CATCCGCCATCCAGTTCATA | 111 | 1.00 | 2.00 |
| *SCD* | NM_001009254 | F: GAGTACCGCTGGCACATCAA  R: CTAAGACGGCAGCCTTGGAT | 103 | 0.96 | 2.00 |
| *PPARG* | AY315429 | F: GCCTCATGAAGAGCCTTCCA  R: CCTTGCATCCTTCACAAGCA | 107 | 0.95 | 1.93 |
| *CEBPA* | AY458599 | F: TGGAGACGCAGCTGAAGGT  R: CCAGTTCGCGGCTCAGTT | 84 | 0.98 | 2.00 |
| *SREBF1* | AY496867 | F: CAATGCCATCGAGAAACGCTA  R: CAGGTCCTTGAGCTCGATGAT | 64 | 0.98 | 2.00 |
| *FADS1* | XM_002699285 | F: CTGCTGTACCTGCTGCACAT  R: ACGGACAGGTGTCCAAAGTC | 161 | 0.99 | 2.00 |
| *FADS2* | NM_001083444 | F: TGCCAACTGGTGGAACCATCGC  R: GCGGCCCGATCAGGAAGAAGTAC | 189 | 0.98 | 1.99 |
| *ELOVL5* | EU747336 | F: TGCTTCAGTTTGTGCTGACC  R: TGGTCCTTCTGGTGCTCTCT | 187 | 0.98 | 2.00 |
| *β-actin* | U39357 | F: CTCACGGAGCGTGGCTACA  R: GCCATCTCCTGCTCGAAGTC | 107 | 0.97 | 1.99 |
